# Silylated biomolecules: Versatile components for bioinks

**DOI:** 10.3389/fbioe.2022.888437

**Published:** 2022-10-11

**Authors:** Titouan Montheil, Matthieu Simon, Danièle Noël, Ahmad Mehdi, Gilles Subra, Cécile Echalier

**Affiliations:** ^1^ IBMM, University Montpellier, CNRS, ENSCM, Montpellier, France; ^2^ ICGM, University Montpellier, CNRS, ENSCM, Montpellier, France; ^3^ IRMB, University Montpellier, INSERM, CHU, Montpellier, France

**Keywords:** sol-gel, bioprinting, bioorthogonal reaction, multifunctional hydrogel, hybrid peptide, hybrid oligosaccharide

## Abstract

Physical hydrogels prepared from natural biopolymers are the most popular components for bioinks. However, to improve the mechanical properties of the network, in particular its durability for long-lasting tissue engineering applications or its stiffness for bone/cartilage applications, covalent chemical hydrogels have to be considered. For that purpose, biorthogonal reactions are required to allow the inclusion of living cells within the bioink reservoir before the 3D printing procedure. Interestingly, such reactions also unlock the possibility to further multifunctionalize the network, adding bioactive moieties to tune the biological properties of the resulting printed biomaterial. Surprisingly, compared to the huge number of studies disclosing novel bioink compositions, no extensive efforts have been made by the scientific community to develop new chemical reactions meeting the requirements of both cell encapsulation, chemical orthogonality and versatile enough to be applied to a wide range of molecular components, including fragile biomolecules. That could be explained by the domination of acrylate photocrosslinking in the bioprinting field. On the other hand, proceeding chemoselectively and allowing the polymerization of any type of silylated molecules, the sol-gel inorganic polymerization was used as a crosslinking reaction to prepare hydrogels. Recent development of this strategy includes the optimization of biocompatible catalytic conditions and the silylation of highly attractive biomolecules such as amino acids, bioactive peptides, proteins and oligosaccharides. When one combines the simplicity and the versatility of the process, with the ease of functionalization of any type of relevant silylated molecules that can be combined in an infinite manner, it was obvious that a family of bioinks could emerge quickly. This review presents the sol-gel process in biocompatible conditions and the various classes of relevant silylated molecules that can be used as bioink components. The preparation of hydrogels and the kinetic considerations of the sol-gel chemistry which at least allowed cell encapsulation and extrusion-based bioprinting are discussed.

## Introduction

In extrusion-based bioprinting, a formulation of cells and materials suitable for 3D printing, called bioink, is loaded into a cartridge and extruded through a nozzle as filaments. (Groll et al., 2018; Zhang et al., 2021). The movement in x,y,z of the printhead and/or the building platform allows to deposit the filaments on the platform according to the intended design and thus build 3D structures layer by layer. The extruded filaments should retain their shape for the printed construct to match the original computer design. Soft hydrogel-based inks are often preferred to prevent excessive shear stresses that could damage embedded cells during the printing process. However, soft materials are also prone to deformation which may alter shape fidelity. Therefore, it is quite challenging to design bioinks that will ensure extrudability through a small-sized nozzle and shape-fidelity of the construct after deposition while preserving cell viability. (Emmermacher et al., 2020; Schwab et al., 2020).

Collagen, hyaluronic acid, alginate and many other high molecular weight biopolymers can readily form physical hydrogels in aqueous solutions. Therefore, they are convenient and easy-to-handle bioink precursors. (Benwood et al., 2021). However, physical hydrogels are stabilized by weak and reversible interactions such as hydrogen bonds, ionic interactions or hydrophobic association. As a consequence, they often suffer from poor mechanical properties and fast degradation rate. On the other hand, creating covalent bonds between precursors results in chemical hydrogels; it allows to tune stiffness and degradability as well as to functionalize the network. Noteworthy, special attention has to be paid to the crosslinking chemistry. The presence of live cells in the bioink enforces constraints in terms of solvent, pH, temperature and reagents. Chemoselective and biorthogonal reactions, i.e., reactions that can occur in living systems without modifying biomolecules or interfering with biological processes, are required to preserve cell viability. (Prescher and Bertozzi, 2005; Valot et al., 2019a; Echalier et al., 2019).

Selected examples of cross-linking reactions used for extrusion-based bioprinting are provided hereafter and summarized in Table 1. Some groups have exploited Michael addition for the preparation of bioinks. For instance, Yan *et al.* described a bioink based on thiolated gelatin crosslinked with a bifunctional maleimide polyethylene glycol (PEG). (Yan et al., 2018). Noteworthy, their network was further stabilized by the introduction of amphiphilic peptides. Cellulose-based bioinks were obtained by Copper-catalyzed Azide-Alkyne Cycloaddition (CuAAC) between azido-hydroxyethyl cellulose and propargyl carboxymethyl cellulose. (Mohamed et al., 2020). Hull et al*.* developed a versatile bioink strategy using the strain-promoted version of the azide-alkyne cycloaddition (SPAAC). (Hull et al., 2021). They introduced dibenzocyclooctyne groups onto gelatin, hyaluronic acid, elastin-like protein and polyethylene glycol and crosslinked them by addition of a diazide-PEG or a tetra-bicyclononyne-PEG. The chemoselective formation of dynamic covalent bonds was also used in bioinks. Burdick and coworkers mixed hyaluronic acid modified with either hydrazide or aldehyde groups to form a bioink with hydrazone crosslinks. (Wang et al., 2018). Mueller et al. recently reviewed click chemistry hydrogels for bioprinting and acknowledged that, to date, relatively few examples of bioinks crosslinked by click chemistry have been reported. (Mueller et al., 2022). Indeed, the bioink field is largely dominated by photocrosslinking. (Pereira and Bártolo, 2015; Lim et al., 2020).

**TABLE 1:** Comparison of sol-gel bioinks with photocrosslinked and click chemistry-based bioinks. One representative reaction was considered for each crosslinking strategy.

Crosslinking strategy	Sol-gel	Photocrosslinking	Click chemistry
Representative reaction	Siloxane formation	Chain-growth photopolymerization	SPAAC
Representative reactive groups	Triethoxysilanes	Acrylates	BCN + azide
Functionalizing reagents	Low cost	Low cost	Expensive (alkyne)
Multicomponents bioinks	Possible	Possible	Limited due to complementary reactive groups
3D printing technique	Extrusion	Extrusion	Extrusion
		Stereolithography	
		Inkjet	
Reaction rate	Slow	Fast	Medium
Bioink stability	Viscosity increases with time	Stable in the dark	Stable until complementary building blocks are mixed
Biocompatibility limitations	Catalyst	Photoinitiator	—
		Photodamages	
		Reactive oxygen species	
Other limitations	—	—	Hydrophobicity of BCN might limit water solubility of building blocks

Photocrosslinking refers to the formation of covalent crosslinks initiated by light irradiation in the presence of a photoinitiator. The photoinitiator absorbs the light and dissociates into free-radical reactive species. These species react with chemical groups onto bioink precursors, triggering their free-radical polymerization. Depending on the chemical groups chosen, the polymerization can proceed through a chain-growth or a step-growth mechanism. Acrylates and methacrylates are the most popular functionalizing groups, they can undergo chain-growth polymerization. Gelatin methacryloyl (GelMA) is one of the most widely used photocrosslinkable precursor for bioinks. (Yue et al., 2015; Ying et al., 2018). Methacrylated hyaluronic acid (HAMA) can also be obtained and was used in combination with GelMA for several applications including 3D printed heart valve conduits. (Duan et al., 2014). Photocrosslinking has been widely adopted by the bioprinting community because it provides spatial and temporal control over the gelation. Photodamages and the formation of reactive oxygen species are limiting the application of UV-photocrosslinking. (Klak et al., 2020; Huh et al., 2021). However, considerable efforts have been made to develop cytocompatible visible light photoinitiators, that are better-tolerated by a broad range of cell lines. (Bryant et al., 2000).

The sol-gel process has recently emerged as a crosslinking chemistry for bioinks. By showing that different types of (bio)molecules and (bio)polymers can be silylated, crosslinked *via* the sol-gel process under biocompatible conditions and 3D printed by extrusion, this review explores the potential of the sol-gel to become an alternative strategy to photocrosslinking in extrusion bioprinting.

## Sol-gel inorganic polymerization

The sol-gel process is an inorganic polymerization (Sanchez et al., 2005) which involves organometallic molecular precursors, reacting together to form a metal oxide network. A wide range of metal oxides and metal halides can be used as precursors, but due to several similarities between silicon and carbon, silicon alkoxides are the compounds of choice for the sol-gel process and have been used in most studies. (Montheil et al., 2018). Examples depicted in the rest of this section are thus focusing on silicon derivatives.

The sol-gel process is characterized by two reactions: 1) hydrolysis of silicon alkoxides into silanols (SiOH), followed by 2) condensation of silanols and/or silicon alkoxides to form siloxane bonds (Si-O-Si). During these steps, the tetravalence of silicon allows the linkage of several partners and the creation of crosslinking nodes. These covalent bonds lead to a stable three-dimensional network.

If the formation of siloxane bonds may be achieved in a wide range of experimental conditions, the process may also occur in water, at ambient temperature. By-products are most of the time water and low molecular weight alcohol. Furthermore, the sol-gel process is a chemoselective and bio-orthogonal reaction meaning that in such conditions, silanols do not react with any other organic functional groups (i.e.*,* amines, carboxylic acids, alcohols, phenols, imidazoles, thiols, etc.) displayed by biomolecules. It allows the incorporation of biological species (for instance living cells) before the reaction starts. These features make it a biocompatible cross-linking method attractive for bioinks.

**FIGURE 1:**
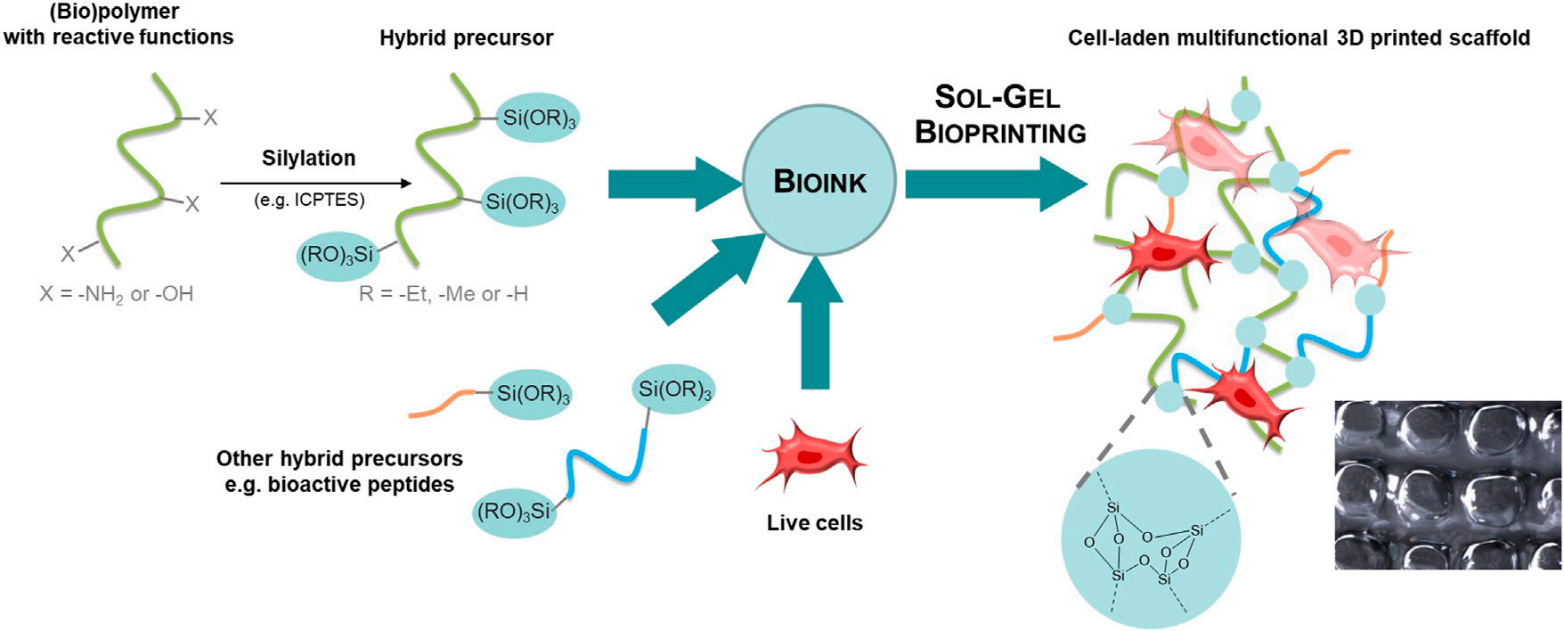
The sol-gel chemistry applied to bioprinting. Hybrid precursors are obtained by functionalization of synthetic or natural polymers, peptides or small organic molecules with alkoxysilane groups using a silylation reagent such as the isocyanatopropyltriethoxysilane (ICPTES). Hybrid precursors and live cells are mixed together in the desired ratio to provide the bioink. The sol-gel process occurs during extrusion-based bioprinting. The 3D printed scaffold encapsulates cells into a covalent organic-inorganic 3D network.

When an organic molecule is linked covalently to a silicon alkoxide through a non-hydrolysable bond (typically a stable C-Si bond), an organic-inorganic precursor is obtained and described as hybrid precursor. The sol-gel chemistry can be used to crosslink hybrid precursors and obtain hybrid materials (Figure 1). Interestingly, several hybrid precursors can be mixed and react together during the sol-gel process, which allows the functionalization of the network during its formation and yields tailor-made multifunctional scaffolds.

Hybrid precursors are obtained by functionalization with a silylation reagent such as 3-isocyanatopropyltriethoxysilane (ICPTES) or 3-glycidyloxypropyltriethoxysilane (GPTES). Successful silylation and isolation of the hybrid precursor require solubility in organic solvent and a nucleophile group on the precursor. In case multiple reactive groups are present, the functionalization can be chemoselective (i.e., functionalization of amines in the presence of alcohols) or may require protecting groups. The reader is referred to reported silylation protocols. (Montheil et al., 2020a). Another requirement for sol-gel bioinks is water-solubility of the silylated molecules since the sol-gel process will start with dissolution of hybrid precursors in aqueous media.

**FIGURE 2:**
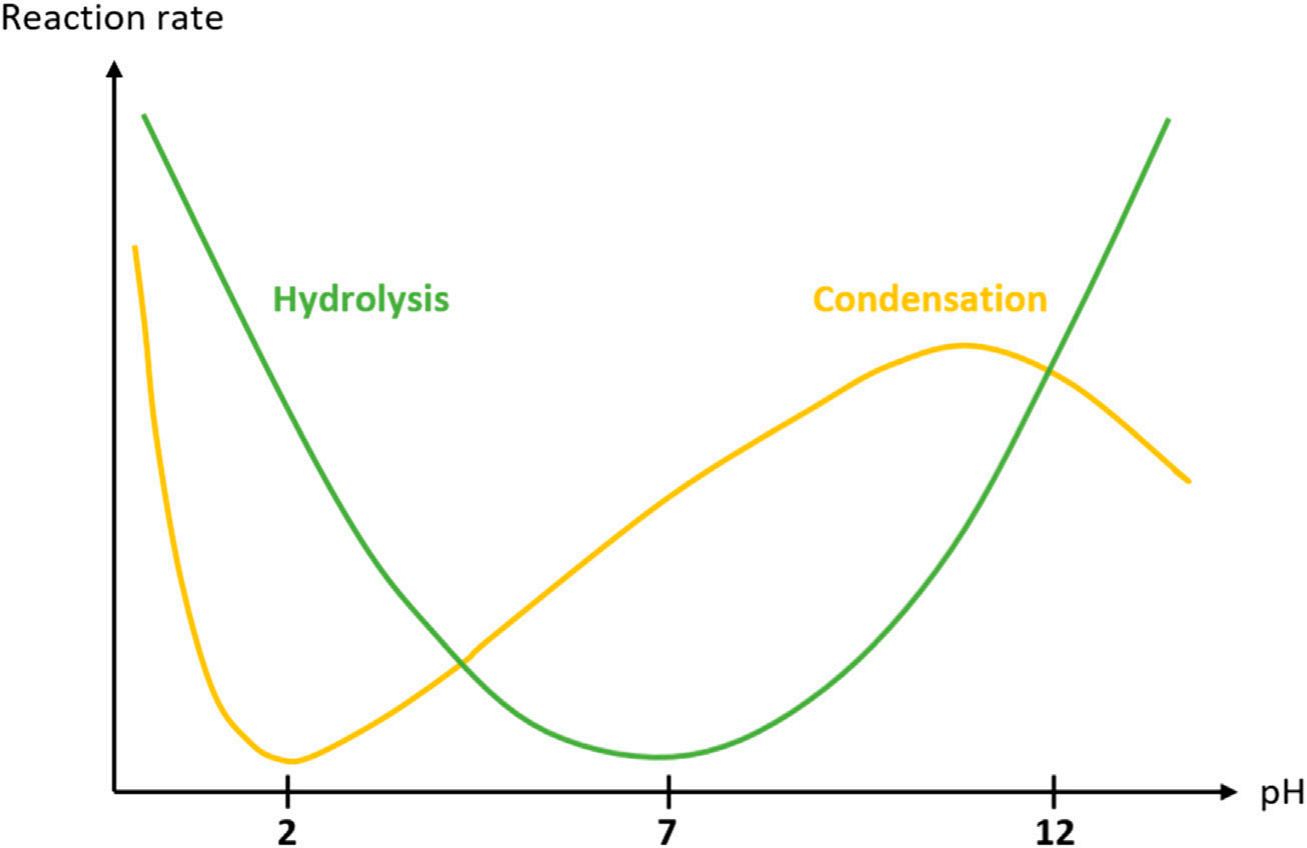
Reaction rate of tetraethylorthosilicate (TEOS) hydrolysis (green curve) and condensation (yellow curve) as a function of pH. (Brinker and Scherer, 1990). Similarly, hydrolysis and/or condensation of trialkoxysilyl groups can be catalyzed by acids, bases but also nucleophiles.

In the sol-gel process, both hydrolysis and condensation kinetics are pH dependant. (Brinker and Scherer, 1990). Briefly, hydrolysis is favored at acidic and basic pH, while condensation is fast at very low pH, decreases dramatically at pH 1.5-2, to finally increase gradually and reach a maximum at pH 10–11 (Figure 2). For the crosslinking to proceed at physiological pH in the presence of cells, two strategies are possible: on the one hand, it is possible to trigger the sol-gel process at extreme pH for fast hydrolysis and neutralize the solution during the sol-gel polymerization before encapsulation of cells; on the other hand, a nucleophile catalyst can be used to conduct the entire sol-gel process at physiological pH. Both strategies are explained below.

Weiss and co-workers took profit of the first stategy to prepare hydrogels based on hybrid hydroxypropyl methyl cellulose (HPMC). (Vinatier et al., 2005; Mathieu et al., 2012; Liu et al., 2014; Viguier et al., 2016; Zhang et al., 2016; Rederstorff et al., 2017; Boyer et al., 2018). As an example, they described an hybrid HPMC hydrogel for cell encapsulation of rabbit chondrocytes. (Vinatier et al., 2009). Nonetheless, the method used for the introduction of silane groups (silylation) and for hydrogel preparation did not allow the prior preparation of a bioink at physiological pH. Indeed, a premature and uncontrolled condensation between alkoxysilanes occurred during the silylation process. As a consequence, the hybrid precursor was not soluble at physiological pH. Thus, dissolution had to be performed at basic pH (>12), this favoured hydrolysis, and a neutralization step was necessary before cell addition.

Recently, our team developed a wide range of silylated components, fully soluble in a cell containing buffer, at physiological pH to obtain hybrid hydrogels (see part 3). This new approach avoids changes of pH and guarantees a cell-friendly environment during all the process.

Since kinetics of hydrolysis and condensation at neutral pH are, respectively, dramatically low and medium low (Figure 2), a nucleophilic catalyst can be used to speed up the process while keeping the pH to neutrality. Sodium fluoride (NaF) is extensively applied for that purpose, (Mehdi et al., 2005), catalyzing both hydrolysis and condensation. (Pope and Mackenzie, 1986; Brinker, 1988; Winter et al., 1988; Tilgner et al., 1995). However, sodium fluoride appears to be toxic for cells at a molarity of 24 mM, which is usually required for efficient catalysis. (Tsutsui et al., 1984; Jeng et al., 1998; Khalil and Da’dara, 1994; Song et al., 2002; Zeiger et al., 1993).

To unlock this problem of toxicity while conserving an efficient catalytic behavior, more than 50 biocompatible catalysts were screened as non-toxic alternatives to NaF, compatible with both sol-gel process and bioink requirement. (Valot et al., 2019b). The effectiveness of fluoride remained unmatched. However, it was found that amino acids, glycine in particular, could be used as a highly efficient co-catalyst, speeding up the gelation while keeping the concentration of fluoride below 2.5 mM, at which cell toxicity was negligible (Table 2).

**TABLE 2:** Examples of sol-gel catalysts.

Sol-gel catalyst	Gelation time	Biocompatibility	References
NaF 3 mg/ml	2.25 h for 10 wt% PEG-Si in DPBS (pH 7.4)	Cytotoxic	Valot et al. (2019b)
NaF 0.1 mg/ml	53 h for 10 wt% PEG-Si in DPBS (pH 7.4)	Biocompatible	Valot et al. (2019b)
NaF 0.1 mg/ml + glycine 10 mg/ml	17.5 h for 10 wt% PEG-Si in DPBS (pH 7.4)	Biocompatible	Valot et al. (2019b)
0.2 M NaOH (hydrolysis) followed by 0.26 M HEPES (condensation)	No gelation in NaOH.	Hydrolysis conditions are cytotoxic, requires neutralization before cell encapsulation	Vinatier et al. (2005)
	0.5 h after addition of HEPES for 3 wt% Si-HPMC in DMEM + FCS		

The cytocompatibility of the approach was demonstrated by embedding primary mouse mesenchymal stem cells in 10 wt% bis-silylated PEG hydrogel containing 0.1 mg/ml of NaF and 10 mg/ml of glycine, in cell-friendly conditions (37°C in DPBS). (Valot et al., 2019b). Thereafter, these biocompatible catalyst conditions were exploited to describe the first example of cell-containing hydrogel scaffold obtained by 3D bioprinting using the sol-gel process. (Montheil et al., 2020b). (See part 3.3).

As the sol-gel process occurs, the viscosity of the solution increases. The measure of the viscosity as a function of time during the gelation appears as a key parameter to define the printability window. This window corresponds to a time lapse where the extrusion of the hydrogel through the syringe is optimal. Before this point, the hydrogel is unable to support itself after extrusion; after the printability window, the hydrogel is too stiff and irregular, broken pieces of gels are extruded. Note that this printability window has to be determined experimentally and varies depending on the type of hybrid precursor used, their concentration, the silylation rate, the media, the catalyst, pH and temperature. As expected, the reaction rate increases with concentration and temperature, but also with the complexity of the medium. It is believed that complex cell culture media contain nucleophiles that can co-catalyze the sol-gel process. Interestingly, addition of cells to the ink also speeds up the sol-gel process. (Valot et al., 2019b).

**FIGURE 3:**
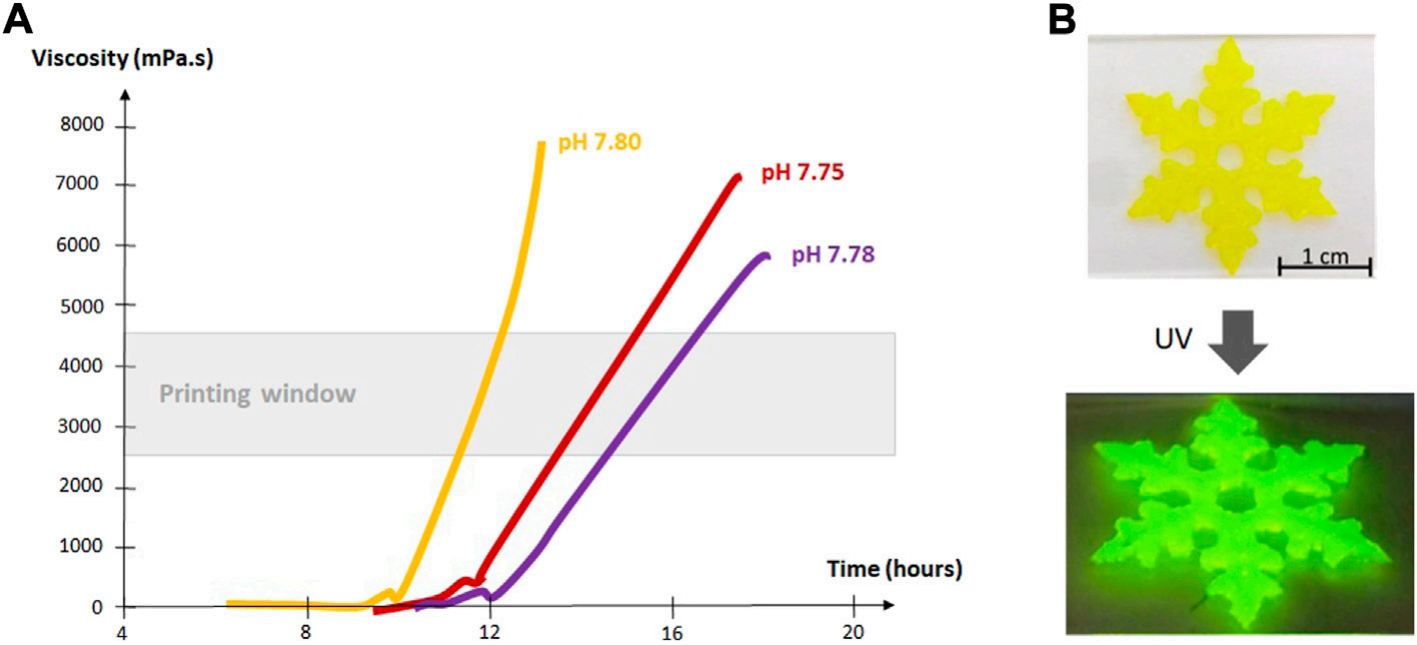
**(A)** Viscosity of hybrid PEG hydrogels in chondrogenic medium (yellow curve), proliferative medium (red curve) and DPBS (purple curve) (37°C, 0.1 mg/ml NaF, 143 mM glycine). (Valot et al., 2019b).The printability window was determined by extrusion tests run in parallel with viscosity measurements. The bioink was deemed printable when a continuous filament could be extruded and could maintain its shape after extrusion. **(B)** Images of a 3D printed scaffold made of 10 wt% bis-silylated PEG hydrogel containing silylated fluorescein.

Figure 3A shows an example of evolution of the viscosity as a function of time for hybrid PEG hydrogels in different media, and the determination of the printability window. If the printability window is well defined, the relative slowness of the sol-gel process can be advantageous for bioinks as it allows printing for extended periods of time. Cells are added into the precursor solution before bioprinting, preferably when the solution is already viscous enough to prevent sedimentation. Small changes in temperature and addition of glycine can be used as a tuner to slow down or speed up the gelation, depending on the requirements of 3D printing.

The trial-and-error process can be reduced if the rheological properties of the ink are well characterized. In addition to viscosity, the elastic modulus, density, surface tension, flow behavior index, consistency index and critical yield stress are essential parameters to define printing parameters. Recently, A. F. Bonatti et al. developed a predictive model to assess printability by extrusion. (Bonatti et al., 2021). The authors proposed a set of rheological experiments to characterize the ink and input relevant data into the mathematic model. This tool has not been applied to sol-gel bioinks yet. It could reduce significantly the experimental printability studies but would need to take into account the change of rheological behavior of the inks over time.

**FIGURE 4:**
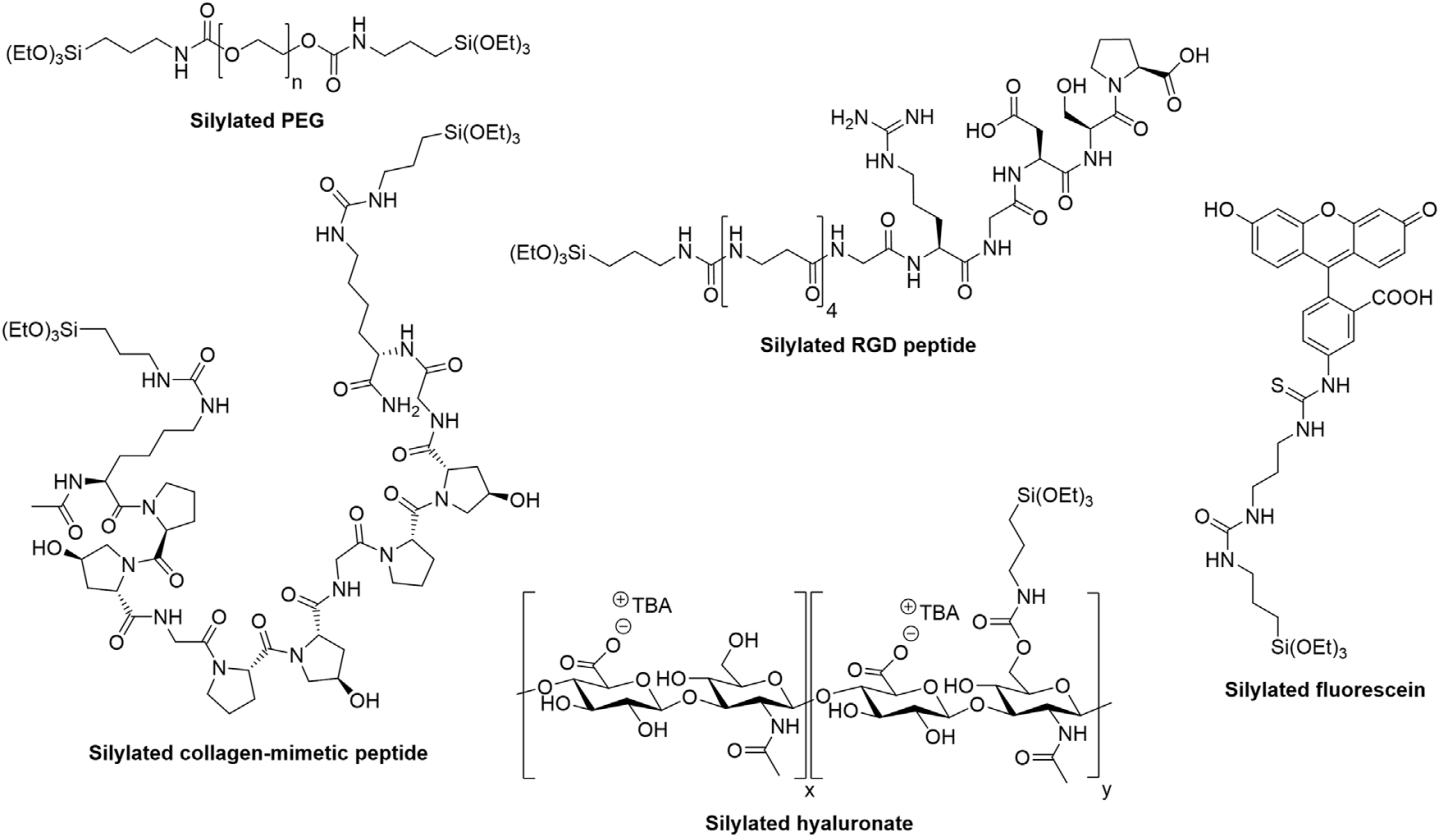
Examples of hybrid precursors that can be used to form and/or functionalize hybrid hydrogels *via* the sol-gel process.

The studies described above (Valot et al., 2019b; Montheil et al., 2020b) paved the way to a better understanding and application of the sol-gel inorganic polymerization for 3D bioprinting. Although only three examples of sol-gel bioinks have been reported to date, the next section presents the numerous silylated (bio)molecules that were synthesized and demonstrates the broad applicability of the silylation reaction. The accessibility of silylated precursors together with the biocompatibility of the crosslinking process suggest a promising development of the sol-gel bioinks in the coming years. Selected hybrid precursors used for the preparation of hydrogels are presented below (Figure 4). As explained above, they can be mixed to obtain tailor-made hybrid materials.

## Bioink silylated components

### Synthetic polymers

Synthetic polymers can be produced in large scale with virtually no batch-to-batch variability. Combined with their ease of modification, it makes them highly attractive precursors for the preparation of materials. Polyethylene glycol (Figure 4) and Pluronic F127 were the first silylated polymers used to prepare hydrogels. (Jo and Park, 1999). They were modified with 3-isocyanatopropyltriethoxysilane and engaged in the sol-gel process, which was not conducted under biocompatible conditions. The procedure involved ethanol, an acidic pH and solvent evaporation.

Recently, Alves et al. reported silylated PEG hydrogels functionalized with an amyloid-like peptide. (Decandio et al., 2020). Their preparation protocol included the use of DMSO, high concentration of sodium fluoride and refrigeration which is not compatible with cell encapsulation. Although the authors did not demonstrate printability, the hydrogels showed thixotropic and shear-thinning behavior.

In earlier work, we went one step further and demonstrated printability of silylated PEG hydrogels by extrusion. (Echalier et al., 2017a). The evolution of viscosity over time was studied and a printability window of a couple of hours was defined for neat deposition of the hydrogel. Noteworthy, the sol-gel process was catalyzed with a high concentration of sodium fluoride, incompatible with cell encapsulation.

Other examples of silylated synthetic polymers include silylated lactones, silylated poly (amido amine) dendrons and silylated star-poly (lactide). (Kricheldorf et al., 2006; Onoshima and Imae, 2006; Arsenie et al., 2020). However, none has ever been used for bioprinting.

Synthetic polymers are considered bioinert in the sense that they are usually well-tolerated by cells but do not elicit an inflammatory response. Therefore, functionalization with bioactive molecules such as peptides derived from the extracellular matrix is required to support cell adhesion and proliferation. (Zhu, 2010).

### Peptides

The bioactivity and versatility of peptides have been largely exploited for several decades in biomaterials and, more recently, in bioinks. (Boyd-Moss et al., 2017). However, synthesis and isolation of silylated peptides were first studied by our group in 2013. (Jebors et al., 2013a). Dimethylhydroxysilyl and triethoxysilyl groups are usually introduced on the N-terminus of the peptide or on a lysine side-chain, either during solid-phase peptide synthesis or in solution after peptide synthesis, in particular through the use of silylated isocyanate derivatives, which react with primary amines. By playing with protecting groups, it is possible to selectively functionalize a peptide only at the desired positions. (Montheil et al., 2020c). The synthesis is well-controlled, highly reproducible and leads to structurally-defined hybrid peptides with no batch-to-batch variability.

Silylated peptides can be used both alone as main network component of bioinks and in combination with other silylated building blocks for functionalization during bioprinting. Silylated collagen-mimetic peptides containing the repeated triplet Pro-Hyp-Gly are so far the only reported peptides that were used as main network components (Figure 4). (Echalier et al., 2017b; Valot et al., 2021) They formed hydrogels at 6–10 wt% in physiological media depending on the number of Pro-Hyp-Gly repeats and provided a chondroconductive environment to embedded mesenchymal stromal cells when cultured in chondroinductive medium. Interestingly, mixing mono-silylated peptides with bifunctional ones allowed fine-tuning mechanical properties towards more elastic hydrogels. Nevertheless, the printability of these collagen-inspired hydrogels has still to be demonstrated.

Alternatively, silylated peptides can be used as additives during the sol-gel process to provide additional functionality, in particular biological activity. The motif Arginine-Glycine-Aspartic acid (RGD) is probably the most widely used peptide sequence in the biomaterial field. (Hersel et al., 2003; Bellis, 2011). Recognized by cell integrins, it promotes adhesion of numerous cell types. A silylated version of an RGD peptide was mixed with silylated PEG to prepare cell-adhesive 3D printable hydrogels (Figure 4). (Echalier et al., 2017a) The NaF concentration used to catalyze the sol-gel process in this work was not suitable for cell encapsulation but the NaF-glycine biocompatible catalysis reported more recently could easily be applied to these hydrogels and enable their bioprinting. (Valot et al., 2019b). Noteworthy, the synthesis of a silylated cyclic RGD peptide was also described. (Ciccione et al., 2016).

In another work, antibacterial hydrogels were obtained by functionalization of PEG-based hydrogels with a silylated cationic peptide displaying antibacterial properties. (Echalier et al., 2016a). The materials were able to inhibit or strongly reduce the growth of major pathogens involved in healthcare associated infections. Although not investigated, the printability of these hydrogels is expected to be similar to RGD-PEG hydrogels described above. (Echalier et al., 2017a).

Alves and co-workers reported a silylated amyloid-like peptide for the preparation of amyloid fibril hydrogels. (Decandio et al., 2020). The peptide was silylated with ICPTES on solid support before cleavage with TFA in the presence of water. Although it was not investigated in the paper, such cleavage conditions should hydrolyze triethoxysilyl groups and might lead to premature condensation resulting in inhomogeneous distribution of the peptide within the hydrogel.

Silylated peptides can also be incorporated to tune the degradation of biomaterials. Indeed, enzyme-sensitive peptides functionalized with at least two silyl groups can be used as cross-linkers. MMP13-sensitive peptide-based materials were recently developed by our group for application in biosensing. (Sandoval, 2021). In the context of regenerative medicine, enzyme-cleavable peptides can ensure that the implanted scaffold degradation will match the production of extracellular matrix by encapsulated cells. (Lutolf et al., 2003).

Other examples of silylated peptides include peptides with biological activity (anti-angiogenic, (Ciccione et al., 2016; Maurel et al., 2021), regulatory, (Jebors et al., 2015; Echalier et al., 2016b), antibacterial (Masurier et al., 2018)), catalytic activity (Jebors et al., 2013b) or enzyme-responsive degradation. (Maggini et al., 2016). Although none of these has ever been used for bioprinting, there is in theory no limitation that could prevent their incorporation in bioinks, except perhaps limited aqueous solubility.

### Proteins

Recently, this silylation method was also successfully transposed to proteins. Proteins are challenging biomolecules to work with. Indeed, protein’s function is usually related to its tertiary structure acquisition in a physiological environment. Obviously, the alkoxysilane grafting step performed at basic pH and in organic solvent is not compatible with the maintenance of the protein 3D structure. To tackle this limitation, the silylation step was performed on an already partially hydrolyzed and unstructured protein: gelatin.

Gelatin is a water-soluble protein derived from collagen. It is obtained after the partial hydrolysis (acidic or alkali) of collagen and, as a consequence, shares really close biological properties with this latter. (Gorgieva et al., 2011). This is of particular interest for cell culture application as collagen is the most abundant protein of the extracellular matrix.

Nowadays, the most commonly used bioink in tissue engineering is the methacryloyl gelatin (GelMA). GelMA is obtained through the reaction of methacrylic anhydride with the primary amine of the lysine’s amino acid side chain of gelatin. (Van Den Bulcke et al., 2000). Under UV exposure and in presence of a photo-initiator, the acrylate group will be crosslinked creating a chemical network that will increase the mechanical properties of gelatin and thus allow cell encapsulation and cell culture at 37°C for several weeks. This bioink has been widely used in many 3D printing and bioprinting applications. (Yue et al., 2015). Cells are embedded into the viscous GelMA solution in combination with a photo-initiator then the mixture is 3D printed in a layer-by-layer manner. Between each layer UV exposition is performed to induce the photopolymerization reaction resulting in an increase of the hydrogel’s stiffness. However, the cytotoxicity induced by this process is still under debate. Indeed, UV exposition and free radical generation lead to DNA damage and reactive oxygen species generation that can be deleterious for cells. (Pahoff et al., 2019; Lee et al., 2020).

In order to offer an alternative to GelMA, a silylated gelatin-based bioink was successfully developed. (Simon et al., 2022). Gelatin was modified with triethoxysilyl groups according to the previously described chemistry and the new hybrid biopolymer was called GelmSi. The silylation was specifically performed on the free amino groups of lysine side chains. This corresponded to a silylation rate of 4% of the total amino acids. The gelation process was triggered during the formation of the polysiloxane network by bringing together the gelatin chains, promoting the establishment of weak interactions between them (van der walls interactions and hydrogen bonds). At similar gelatin concentration, this hydrogel displayed rheological properties close to GelMA and was able to encapsulate adipose-derived mesenchymal stromal cells with 90% viability after 7 days at 37°C (Table 3).

**TABLE 3:** Comparison between GelMA and GelmSi hydrogels.

	% (w/V)	DoF	Chemistry	Reticulation initiation	G’ (kPa)	Cell viability	References
GelMA	10	82–85%	MAA	0.5% w/w of Irgacure 2,959, UV (10 mW cm^−2^ at 365 nm, 60–120 s)	26	>90% after 7 days (hDF)	Shie et al. (2020)
GelMA	20	100%	MAA	0.5% w/w of Irgacure 2,959, UV (3.5 mW cm^−2^ at 365 nm, 5 min)	30	90% after 7 days (Huh7.5)	Zhu et al. (2019)
GelmSi	10	98%	ICPTES	NaF 0.1 mg/ml + glycine 10 mg/ml, 3 h	16	90% after 7 days (ASCs)	Simon et al. (2022)

Methacrylic anhydride (MAA), Degree of Functionalization (DoF), storage modulus (G′), human Dermal Fibroblasts (hDF), Human hepatocellular carcinoma cells (Huh7.5), Human Adipose-derived mesenchymal Stromal Cells (ASCs).

Interestingly at the beginning of the sol-gel transition, the GelmSi behaves similarly to gelatin and GelMA and can be 3D printed using similar printing parameters. (Wang et al., 2017). In order to promote the formation of a physical gel of gelatin, the bed of the 3D printer was cooled at 10°C. After 3 hours, the siloxane covalent network was fully formed and the temperature could be increased up to 37°C, without affecting the integrity of the 3D printed scaffolding.

The possibility to play with the parameters favoring either the formation of a physical gel or a chemical gel makes this modified biopolymer particularly interesting for 3D bioprinting applications. GelmSi could also be mixed with other biopolymers as it has already been done for gelatin or GelMA with alginate and/or fibrinogen to further improve its biological properties. (Afewerki et al., 2019). Of course as explained previously, by taking advantage of the biorthogonality of this chemistry, complex networks can be created by mixing different silylated biomolecules adapted to a specific organ type. The possibilities of development of this silylated biopolymer for 3D bioprinting are numerous.

Overall, GelMA and GelmSi displayed close biological properties. These results suggest that biological properties are mainly driven by the type of building block used with little influence of the crosslinking chemistry. It is expected that crosslinking chemistry will rather affect mechanical properties and degradation.

To date, gelatin is the only reported example of silylated protein. A silylation strategy in aqueous solution is still to be developed in order to preserve the 3D structure of proteins during the modification. The challenge of aqueous silylation lies in avoiding the hydrolysis and premature condensation of alkoxysilane groups during the silylation reaction. Overcoming this challenge would broaden the library of silylated biomolecules.

### Oligosaccharides

Another interesting class of components for the design of bioactive materials are polysaccharides. Indeed, they are most of the time biocompatible and display interesting biological properties (cell adhesion, cell differentiation, biodegradability, resorption,...). (Pina et al., 2015). Alginate, gellan gum, agarose and hyaluronic acid were used for bioprinting with variable occurrences. (Oliveira et al., 2010; Ho et al., 2017; Mondschein et al., 2017; Valot et al., 2019a). Mainly used to create physical hydrogels, polysaccharides can also be covalently cross-linked to create chemical hydrogels. Such materials display higher stability and improved mechanical properties.

Hybrid materials obtained from polysaccharides through sol-gel reaction have been described for chitosan, (Shirosaki et al., 2005; Connell et al., 2014; Wang et al., 2015; Pipattanawarothai et al., 2017), hyaluronic acid, (Lee et al., 2019), alginate (Hosoya et al., 2004) or HPMC. (Bourges et al., 2002; Vinatier et al., 2005; Rederstorff et al., 2017). Nonetheless, all these materials and protocols were not suitable for bioink design because the sol-gel process was not conducted under physiological conditions.

Some example of cell encapsulation within hybrid polysaccharide-based hydrogels for biological applications were described by Weiss and co-workers. (Vinatier et al., 2005; Vinatier et al., 2009; Mathieu et al., 2012; Liu et al., 2014; Viguier et al., 2016; Zhang et al., 2016; Rederstorff et al., 2017; Boyer et al., 2018). These interesting studies present nevertheless some limitations due to successive pH adjustments. First of all, the silylated oligosaccharide precursors were not isolated and may have been contaminated by unreacted silylating reagents. Moreover, premature formation of siloxane bonds occurred during the silylation reaction. (Montheil et al., 2020b). As a consequence, high basic pH values (pH > 12) were required to depolymerize and solubilize the hybrid precursors, and form the silanolate derivatives. Sol-gel inorganic polymerization was triggered by lowering the pH between 8 and 10. Later on, the pH was finally adjusted to 7.4 to allow the addition of cells.

We set up a different sol-gel approach, keeping pH 7.4 all along the process. We first showed that five types of polysaccharides (i.e. HPMC, hyaluronic acid (Figure 4), dextrin, chitosan and pectin) could be silylated, isolated and used as hybrid precursors to prepare hybrid hydrogels. (Montheil et al., 2020b; Montheil et al., 2022). These biopolymers display representative reactive functions on the saccharide units including primary or secondary alcohols, for the three first polysaccharides, primary amine and carboxylic acid groups for chitosan and pectin, respectively.

The key parameter for the design of well-defined hybrid polysaccharide precursors is the monitoring of the silylation ratio, which allows to control the density of reticulation nodes and the resulting mechanical properties of hydrogel network (e.g., the higher cross-linking density, the higher the shear modulus, and the lower the diffusivity within the hydrogel). Moreover, the final precursor must be as pure as possible and the presence of silylating reagent at the end has to be avoided. This can be achieved by repeated precipitation/centrifugation cycles. Purity and silylation degree can be assessed by Nuclear Magnetic Resonance (NMR). Indeed, ^1^H and ^13^C quantitative NMR, ^29^Si NMR and ^29^Si solid state NMR provide a way to ensure that the silylated polysaccharide is obtained as a non-hydrolyzed and uncondensed triethoxysilane. Moreover, these measurements provide a robust way to determine the silylation degree of the hybrid polysaccharide.

After isolating well-controlled hybrid HPMC precursors, the silylation process was extended to the other selected polysaccharides. A silylation degree of about one-third of all the available reactive functions of the polysaccharide was targeted. Once the hybrid polysaccharide precursors were isolated and characterized, the bioink was manufactured by mixing the desired hybrid precursors in PBS buffer containing human mesenchymal stromal cells and adding the sol-gel catalysts (0.1 mg/ml NaF +10 mg/ml glycine). The biocompatibility of the hybrid hydrogels was assessed at day 1 and 7. After 7 days, approximately 70% and 60% of mesenchymal stem cells were still alive within HA and Pectin hydrogel respectively. 3D bioprinting was first optimized with silylated HPMC (on a BioBots 3D device). Simultaneously, the evolution of viscosity was recorded over time and a printability window was defined as a period of time during which viscosity was optimal to extrude a continuous and self-supporting filament of hybrid gel. This study is the first example of 3D printed hybrid bio-ink crosslinked by the sol-gel process. (Montheil et al., 2020b). Our goal is now to apply this technique to the other silylated polysaccharides.

### Small molecules

Synthetic organic molecules such as fluorophores can also be silylated and covalently attached into hybrid hydrogels (Figure 3B, Figure 4). (Echalier et al., 2016a; Maurel et al., 2021) Although they have not been exploited for bioprinting yet, we can envision that silylated fluorophores could facilitate imaging of 3D printed scaffolds and the study of cell-material interactions.

Silylated drugs have also been described and used in prodrug strategies. Xu et al*.* synthesized trialkoxysilyl derivatives of the anticancer drugs camptothecin and doxorubicin. (Xu et al., 2015). The silylated drugs were covalently encapsulated into silica nanoparticles. Glutathione- and pH-responsive bonds were included in the linker between the drug and the silyl groups to enable stimuli-triggered drug release in tumors. A similar strategy could be considered for the controlled delivery of active drugs from 3D printed implantable scaffolds.

## Conclusion and perspectives

The sol-gel process has recently emerged as a crosslinking strategy for bioprinting even though it cannot be temporally and spatially controlled as easily as photoinduced polymerization. Its application to extrusion-based printing requires optimization as the viscosity of the ink varies with the establishment of the siloxane cross-linked network. However, this drawback can be alleviated by adding (hybrid) bioink precursors that can form physical hydrogels to ensure cohesion of the gel immediately after extrusion. Interestingly, the hydrolysis and condensation of alkoxysilane precursors proceed chemoselectively and in a bioorthogonal manner allowing the encapsulation of different cell types. Likely, the main strength of the sol-gel process lies in the unlimited range of molecular components that can be silylated and blended to get the desired bioink composition with the expected biological properties. By playing with the reticulation degree, the biopolymer lengths, and the density of silylated groups, the mechanical properties can also be finely tuned. Other successful strategies applied to other crosslinking methods could be implemented for sol-gel based bioinks including reversible linkages, (Wang et al., 2018), linkers whose structure could be controlled by external stimuli like enzymatic degradation, (Liu et al., 2021), magnetic field or light. (Koetting et al., 2015; Wan et al., 2020). Such features could be used to release bioactive compounds or to allow cell migration.

Besides exploring novel biomimetic compositions that may compete with natural extracts for cell migration, viability and differentiation, the next challenge for sol-gel based bioprinting will be its application to other 3D printing techniques including inkjet and stereolithography.
